# Evaluation of the Accuracy of the Smart Work Injury Management (SWIM) System to Assist Case Managers in Predicting the Work Disability of Injured Workers

**DOI:** 10.1007/s10926-024-10199-7

**Published:** 2024-06-14

**Authors:** Yumiki Y. K. Yeung, Peter Q. Chen, Peter H. F. Ng, Andy S. K. Cheng

**Affiliations:** 1 Department of Rehabilitation Sciences, The Hong Kong Polytechnic University, Hung Hom, Kowloon, Hong Kong; 2 Department of Computing, The Hong Kong Polytechnic University, Hung Hom, Kowloon, Hong Kong; 3 School of Health Sciences, Western Sydney University, Sydney, Australia

**Keywords:** Clinical decision-making support, Prediction, Artificial intelligence, Worker’s compensation, Work disability management

## Abstract

**Purpose:**

Many countries have developed clinical decision-making support tools, such as the smart work injury management (SWIM) system in Hong Kong, to predict rehabilitation paths and address global issues related to work injury disability. This study aims to evaluate the accuracy of SWIM by comparing its predictions on real work injury cases to those made by human case managers, specifically with regard to the duration of sick leave and the percentage of permanent disability.

**Methods:**

The study analyzed a total of 442 work injury cases covering the period from 2012 to 2020, dividing them into non-litigated and litigated cases. The Kruskal–Wallis post hoc test with Bonferroni adjustment was used to evaluate the differences between the actual data, the SWIM predictions, and the estimations made by three case managers. The intra-class correlation coefficient was used to assess the inter-rater reliability of the case managers.

**Results:**

The study discovered that the predictions made by the SWIM model and a case manager possessing approximately 4 years of experience in case management exhibited moderate reliability in non-litigated cases. Nevertheless, there was no resemblance between SWIM’s predictions regarding the percentage of permanent disability and those made by case managers.

**Conclusion:**

The findings indicate that SWIM is capable of replicating the sick leave estimations made by a case manager with an estimated 4 years of case management experience, albeit with limitations in generalizability owing to the small sample size of case managers involved in the study.

**Implications:**

These findings represent a significant advancement in enhancing the accuracy of CDMS for work injury cases in Hong Kong, signaling progress in the field.

## Introduction

Prolonged worker absence from work due to injury is a global concern that has been on the rise in recent years. From 2010 to 2020, the number of days lost due to work-related injuries increased by 42% in 64 surveyed countries, totalling approximately 62.5 million days [1]. From 2010 to 2016, the number of days lost in Hong Kong remained relatively stable at approximately 300,000 days per year [1]. In 2021, there were 30,185 non-fatal occupational injuries in Hong Kong [2]. This phenomenon of enormous loss of days has led to lost wages, lower productivity, and higher permanent disability. In addition, work injuries have created a public health concern [3] and have indicated the need for injury prevention or health and safety reinforcement for workers in the future.

In Hong Kong, protracted waiting periods for medical consultations and rehabilitation interventions, especially with orthopedic specialists at public hospitals, significantly impede the recuperation of injured employees. This delay in obtaining essential medical care aggravates the consequences of injuries. Furthermore, the escalating demand for rehabilitation services exerts pressure on the public healthcare system, complicating the provision of prompt and sufficient assistance to injured workers. In response to these challenges, various rehabilitation initiatives, including the Volunteer Rehabilitation Programme (VRP) [4], the Multi-disciplinary Orthopaedics Rehabilitation Empowerment (MORE) program [5], and the Pilot Rehabilitation Programme for Employees Injured at Work [6], have been established to mitigate these issues. These initiatives prioritize case management and early intervention strategies to guarantee swift and efficient delivery of rehabilitation services.

### Case Management in Hong Kong

Case management has a rich history dating back to 1863 in the United States [7]. In 1995, Martin described case management as “a systematic approach to coordination of services to suitable clients through efforts of assessing providers, treatments, and developing treatment plans which improve quality and efficacy while controlling costs and monitoring outcomes” [8]. The case management model has been effective in reducing the number of lost days, medical costs, and claim costs [9], and importantly, it enhances continuity of case management and promoted efficient reintegration of employees into the workplace [10]. Therefore, over the last 20 years, many employers and insurers in Hong Kong have adopted the case management approach in managing work injury cases to reduce work disability in terms of sick leave days and permanent disability percentage (PD%). In Hong Kong, case management encounters obstacles stemming from a high rate of staff turnover and a deficiency of seasoned case managers. Consequently, this situation leads to novices, lacking formal training or qualifications, managing complex cases.

Case managers play a vital role in estimating reserves for work injury cases. A reserve is defined as funds set aside by an insurance company to meet future obligations as they become due. These obligations include liabilities for unearned premiums and the estimated costs of unpaid claims [10]. Insurance companies earmark reserves to guarantee ample funds are available for policyholders' claims. The precision of these estimates’ hinges on the case manager's competence in evaluating the number of sick leave days and the percentage of permanent disability (PD%). Lack of experience among case managers can compromise the accuracy of reserve estimations and influence the formulation of rehabilitation plans. The distinct reserving guidelines of insurance companies, coupled with the varied quality and experience of case managers, further affect the accuracy of reserves. Additionally, an accurate disability prognosis is pivotal in work injury cases, as it shapes decisions regarding medical interventions, allocation of resources, and the compensation reserved by insurers for such events [11, 12]. An inaccurate estimation of the prospective outcome of an injury can negatively affect the worker's recovery trajectory [13]. Artificial intelligence-powered clinical decision tools for disability forecasting enhance consistency and mitigate the risk of inaccurate medical assessments [14, 15].

### Clinical Decision-Making Support Tools

Clinical decision-making support (CDMS) tools, widely used in various medical areas, have shown potential for improving outcomes such as ambulatory/primary care [16] and genetics healthcare [17]. Nevertheless, further studies are needed to evaluate the results and to determine effective implementation. However, their application in work injury rehabilitation remains limited. A few predictive models have been developed in the industry, but these models have not yet been widely used. Predictive models the Work Assessment Triage Tool (WATT) [18, 19] the gradient boosting machine (GBM) [20] offer potential but need further validation. The Smart Work Injury Management (SWIM) system, developed in Hong Kong, utilizes artificial intelligence to predict return-to-work trajectories and provide advice on medical care and interventions [21, 22]. Unfortunately, there has been no validation study to show its accuracy. Therefore, the objective of this study is to evaluate the accuracy of SWIM as a clinical decision support tool for forecasting work disability in injured workers. By juxtaposing predicted values with estimations made by case managers, the study seeks to gauge SWIM's suitability for industry application and pinpoint its limitations.

## Method

### Designs

**Fig. 1:**
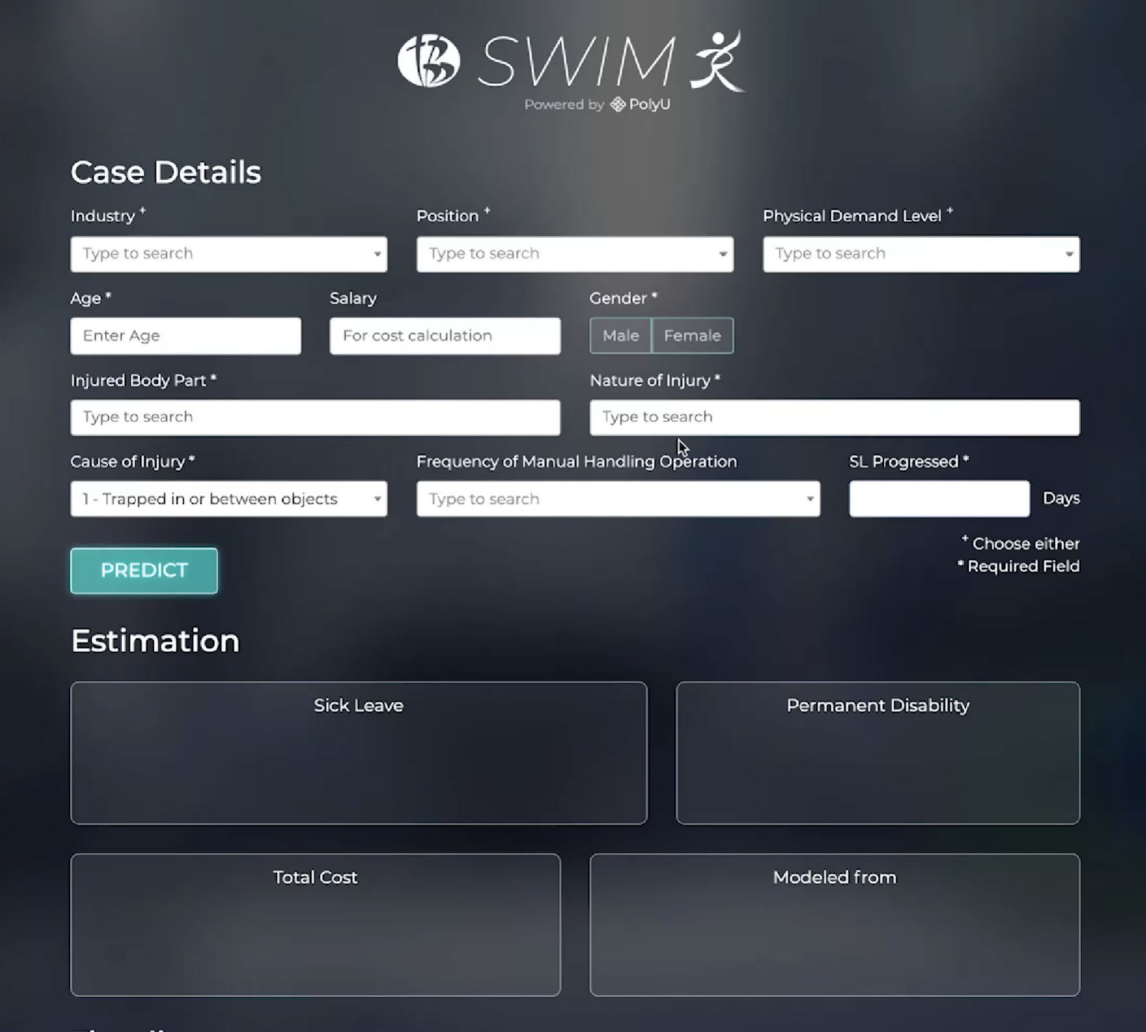
User interface of SWIM

This study used the severity model of the SWIM system (version 1.0) (Fig. 1) to predict sick leave days, PD%, Medical Assessment Board (MAB) days, whether a case requires high-level management (HLM), and return-to-work (RTW) days. The study only used the sick leave days and PD% results for analysis. SWIM requires five mandatory data points to produce a prediction: age, gender, injured body part, nature of injury, and cause of injury. Other optional information that can be inputted includes industry, position, and salary. Physical demand level, alertness, and frequency of manual handling operations are preset and appear on the mandatory data input. These preset fields can be adjusted manually on the basis of the particular situation of a case. In this study, we compared the sick leave days and PD% predictions of the SWIM system with the estimations of three case managers (CMs) with different levels of case management experience and backgrounds: CM-A, a female with 4 years of work injury case management experience and an academic background in marketing; CM-B, a female with 9 years of case management experience and a registered nurse; and CM-C, a female with approximately 20 years of experience and a legal background. CM-B and CM-C were working in the same company at the time of the data collection (Table 1). Since the total number of cases used was 442, only three case managers agreed to participate voluntarily in the study due to the long length of time required for the estimations. Case managers were provided with data for 442 cases in an Excel format, which included an index number starting from 1 to 442, occupation, physical demand level, age at the date of the accident, gender, alertness, injured body part, nature of the injury, cause of injury, and the frequency of Manual Handling Operation for estimating sick leave and PD% (permanent disability percentage). The directive given to the three case managers was to assess each case as if it were newly reported, and to estimate the initial sick leave in months and the PD% that might be awarded following a medical assessment by the Employees’ Compensation (Ordinary Assessment) Board in Hong Kong. No further discussions or surveys were conducted by the case managers.

**Table 1:** Demographic information of case managers

	CM-A	CM-B	CM-C
Gender	Female	Female	Female
Age	35–40	35–40	45–50
Work injury case management experience (years)	4	9	20
Academic background	Marketing	Nursing	Legal
Company	Different from CM-B and CM-C	Same as CM-C	Same as CM-B

### Participants

The data were extracted from the claims database of an insurance company in Hong Kong. A total of 546 claims covering the period between 2012 and 2020 were initially identified; 104 of these cases were not valid and were excluded because they were either fatal cases, cases with no claim form, or cases with insufficient information. The remaining 442 work injury cases (mean age = 45.9; male = 397 and female = 55) were adopted for this study. The data contained the following information about the cases: the demographic information (occupation, age at the date of accident, and gender) of the injured workers (Table 2), information on physical demand level, alertness, injured body part, nature of the injury, cause of injury, frequency of manual handling operations, actual sick leave, and actual PD%. PD% data were available for 267 of the 442 cases, and all cases contained data on the actual sick leave days taken by the injured workers. At the time of the data collection, these cases were at different stages. A total of 332 out of the 442 cases involved employees’ compensation litigation proceedings and 110 were non-litigation cases.

**Table 2:** Demographic and injury information of participants

Variables		Validation data (*n* = 442)	Percentage (%)
1	Age at date of accident (years)	Mean (SD) = 45.9 (11.5)	
2	Sex		
	Female	55	12.400
	Male	387	87.600
3	Occupation		
	Air duct fitter	1	0.2
	Air-conditioning engineer	6	1.4
	Bar bender & fixer	26	5.9
	Bricklayer	1	0.2
	Carpenter	21	4.8
	Cleaner	2	0.5
	Concrete worker	6	1.4
	Construction site worker	142	32.1
	Crane operator	5	1.1
	Curtain wall technician	5	1.1
	Driver	4	0.9
	Electric technician	21	4.8
	Fitter	4	0.9
	Foreman	19	4.3
	Forklift operator	1	0.2
	Formwork worker	10	2.3
	Ganger	1	0.2
	General laborer	23	5.2
	Glass panel installer	1	0.2
	Installation worker	2	0.5
	Landscape attendant	1	0.2
	Leveller	2	0.5
	Machine operator	3	0.7
	Marble worker	2	0.5
	Mason	1	0.2
	Mechanic	1	0.2
	Metal worker	8	1.8
	Nil	27	6.1
	Painter	4	0.9
	Plasterer	13	2.9
	Plumber	13	2.9
	Rigger	24	5.4
	Scaffolder	18	4.1
	Security guard	1	0.2
	Skilled laborer	3	0.7
	Steel worker	6	1.4
	Technician	4	0.9
	Welder	10	2.3
4	Injured body part		
	Abdomen	1	0.2
	Ankle	29	6.6
	Back	72	16.3
	Chest	19	4.3
	Ear	1	0.2
	Elbow	8	1.8
	Eye	13	2.9
	Face	1	0.2
	Finger	67	15.2
	Foot	26	5.9
	Forearm	16	3.6
	Hand/palm	22	5
	Hip	2	0.5
	Knee	17	3.8
	Leg	28	6.3
	Mouth/tooth	3	0.7
	Neck	18	4.1
	Pelvis/groin	2	0.5
	Shoulder	20	4.5
	Skull/scalp	9	2
	Thigh	6	1.4
	Upper arm	6	1.4
	Nil	56	12.7
5	Nature of injury		
	Abrasion	3	0.7
	Amputation	10	2.3
	Burn	3	0.7
	Concussion	1	0.2
	Contusion & bruise	64	14.5
	Crushing	11	2.5
	Dislocation	5	1.1
	Electric shock	2	0.5
	Fracture	147	33.3
	Freezing	1	0.2
	Irritation	1	0.2
	Laceration and cut	21	4.8
	Multiple injuries	12	2.7
	Others	15	3.4
	Puncture wound	3	0.7
	Sprain & strain	91	20.6
	Nil	52	11.8

### Procedures

All mandatory and available optional data were inputted into SWIM. The predicted sick leave days and PD% were recorded for the 442 cases as the experimental group. The same set of data was given to CM-A, CM-B, and CM-C, without knowing the results from SWIM, as the control group. The case managers estimated the sick leave days and the PD% on the basis of the case information provided. Then, the SWIM projected values were compared with the estimations made by the case managers and the actual cases.

The analysis was separated into a group of 110 non-litigated cases and a group of 332 litigated cases. There were 442 cases with data on sick leave days and 273 with PD% data. A litigated case was defined as a case where the injured worker had filed a civil litigation claim against their employer. First, the Shapiro–Wilk test was used to assess the normality of the dataset using Statistical Package for the Social Science (SPSS) version 28 [23]. The test indicated that the sick leave days and PD% data were not distributed normally. Therefore, the Kruskal–Wallis post hoc test with Bonferroni correction was utilized to assess the significant difference between the actual cases, SWIM, and the case managers. The Kruskal–Wallis test is the non-parametric equivalent of a one-way ANOVA and is used for testing whether samples originate from the same distribution [24]. The test was followed by the post hoc Dunn–Bonferroni test to identify the pairwise significant difference from each group by adjusting the alpha level in reducing the type 1 error [25]. Dunn’s test has proved popular over the past two decades, and the test is frequently used with multiple comparison adjustments [24]. Thirdly, the intra-class correlation coefficient (ICC) was selected to test the inter-rater reliability of the predictions across different case managers. ICCs with 95% confidence intervals were calculated using IBM SPSS version 28.0.0.0 (190) based on a mean-rating (*k* = 3), absolute agreement, 2-way mixed-effects model (model 3) [25]. The ICC is a value between 0 and 1, where values below 0.5 indicate poor reliability, values between 0.5 and 0.75 indicate moderate reliability, values between 0.75 and 0.9 indicate good reliability, and any value above 0.9 indicates excellent reliability [26, 27]. Further analysis will be conducted for the distribution of sick leave days and PD% by the nature of injury.

## Results

### Sick Leave Days

#### Kruskal–Wallis Test

The Shapiro–Wilk test showed that the distributions were statistically significantly (*p* < 0.001) non-normal for all the data, including both sick leave days and PD%. Therefore, the Kruskal–Wallis test was used to assess the similarity between the actual data, SWIM predictions, and the estimations produced by the three case managers. The null hypothesis for the Kruskal–Wallis test is that the population medians of the groups are equal. A non-rejection of the null hypothesis signifies that two or multiple groups are significantly similar. Post hoc test with Bonferroni adjustment was adopted for making pair-wise comparisons [24]. The evaluation targeted two distinct groups: (1) non-litigated cases and (2) litigated cases, with the findings displayed in Table 3. For the non-litigated cases, the Kruskal–Wallis test indicated that three pairwise comparisons did not reject the null hypothesis, thereby suggesting that the populations of these pairs were statistically significantly equivalent: (1) CM-A & SWIM (*p* = 0.507); (2) CM-B and the actual data (*p* = 0.109); and (3) CM-C and CM-B (*p* = 0.215). For the litigated cases, only CM-B and CM-C failed to reject the null hypothesis (*p* = 0.153).

**Table 3:** Results of Kruskal–Wallis Test with the post hoc test and Bonferroni correction for sick leave estimation

	Group 1-Group 2	Test statistic	Std. error	Std. test statistic	Sig	Adj. sig.^a^
Non-litigated (*n* = 110; df = 4)	SWIM & CM-C	− 95.145	21.329	− 4.461	< 0.001	0.000
	SWIM & CM-B	− 121.609	21.329	− 5.702	< 0.001	0.000
	SWIM & actual	155.755	21.329	7.302	< 0.001	0.000
	CM-A & SWIM	14.145	21.329	0.663	0.507	1.000
	CM-A & actual	169.900	21.329	7.966	< 0.001	0.000
	CM-B & actual	34.145	21.329	1.601	0.109	1.000
	CM-C & actual	60.609	21.329	2.842	0.004	0.045
	CM-A & CM-B	− 135.755	21.329	− 6.365	< 0.001	0.000
	CM-A & CM-C	− 109.291	21.329	− 5.124	< 0.001	0.000
	CM-C & CM-B	26.464	21.329	1.241	0.215	1.000
Litigated (*n* = 332; df = 4)	SWIM & CM-C	− 318.227	37.122	− 8.573	0.000	0.000
	SWIM & CM-B	− 371.331	37.122	− 10.003	0.000	0.000
	SWIM & Actual	876.318	37.122	23.607	0.000	0.000
	CM-A & SWIM	115.809	37.122	3.120	0.002	0.018
	CM-A & Actual	992.127	37.122	26.726	0.000	0.000
	CM-B & Actual	504.986	37.122	13.604	0.000	0.000
	CM-C & Actual	558.090	37.122	15.034	0.000	0.000
	CM-A & CM-B	− 487.140	37.122	− 13.123	0.000	0.000
	CM-A & CM-C	− 434.036	37.122	− 1.692	0.000	0.000
	CM-C & CM-B	53.104	37.122	1.431	0.153	1.000

^a^Significance values have been adjusted by Bonferroni correction for multiple tests. Each row tests the null hypothesis that the Group 1 and Group 2 distributions are the same. Asymptotic significances (2-sided tests) are displayed. The significance level is 0.050

#### Intraclass Correlation Coefficient (ICC)

Using the ICC, the inter-rater reliability of the SWIM predictions was tested across different case managers for sick leave days (Table 4). The test showed moderate reliability between SWIM and CM-A (ICC = 0.637, *p* < 0.001), CM-A and CM-C (ICC = 0.642, *p* < 0.001), and CM-A and CM-B (ICC = 0.581, *p* < 0.001) for non-litigated cases. CM-B and CM-C demonstrated good reliability for both non-litigated and litigated cases respectively (ICC = 0.837, *p* < 0.001; ICC = 0.845, *p* < 0.001).

**Table 4:** Intraclass correlation coefficient (ICC) calculation using multiple rating, absolute agreement, two-way mixed-effects model for sick leave days

	Average measures	Intraclass correlation^b^	95% confidence interval		F test with true value 0			
			Lower bound	Upper bound	Value	df1	df2	Sig
Non-litigated (*n* = 110; df = 109)	SWIM and CM-A	0.637^c^	0.470	0.751	2.739	109	109	< 0.001
	SWIM and CM-B	0.408^c^	− 0.088	0.662	2.326	109	109	< 0.001
	SWIM and CM-C	0.389^c^	0.015	0.612	1.973	109	109	< 0.001
	CM-C & CM-B	0.837^c^	0.751	0.892	6.607	109	109	< 0.001
	CM-A & CM-C	0.642^c^	0.090	0.827	4.190	109	109	< 0.001
	CM-A & CM-B	0.581^c^	− 0.111	0.810	4.061	109	109	< 0.001
	Actual & CM-A	0.185^c^	− 0.111	0.416	1.414	109	109	0.036
	Actual & CM-B	0.322^c^	0.034	0.528	1.563	109	109	0.010
	Actual & CM-C	0.159^c^	− 0.145	0.393	1.240	109	109	0.131
	Actual & SWIM	0.183^c^	− 0.113	0.414	1.417	109	109	0.035
Litigated (*n* = 332; df = 331)	SWIM and CM-A	0.473^c^	0.331	0.583	1.989	331	331	< 0.001
	SWIM and CM-B	0.173^c^	− 0.060	0.356	1.379	331	331	0.002
	SWIM and CM-C	0.210^c^	− 0.035	0.395	1.438	331	331	< 0.001
	CM-B & CM-C	0.845^c^	0.807	0.875	6.535	331	331	< 0.001
	CM-A & CM-C	0.456^c^	− 0.169	0.724	3.086	331	331	< 0.001
	CM-A & CM-B	0.421^c^	− 0.193	0.706	3.029	331	331	< 0.001
	Actual & CM-A	0.014^c^	− 0.046	0.080	1.055	331	331	0.312
	Actual & CM-B	0.079^c^	− 0.074	0.221	1.229	331	331	0.030
	Actual & CM-C	0.083^c^	− 0.077	0.231	1.252	331	331	0.021
	Actual & SWIM	0.021^c^	− 0.048	0.096	1.080	331	331	0.242

Two-way mixed-effects model where people effects are random and measures effects are fixed

^a^The estimator is the same, whether the interaction effect is present or not

^b^Type An intraclass correlation coefficients using an absolute agreement definition

^c^This estimate is computed assuming the interaction effect is absent because it is not estimable otherwise

### Permanent Disability Percentage

The SWIM system’s predictions on PD% had no similarity across all groups, irrespective of whether the cases were litigated or non-litigated (Table 5). However, the actual data were similar when compared with CM-A (*p* = 0.289) and CM-B (*p* = 0.158) for non-litigated cases and with CM-C (*p* = 0.324) for litigated. The PD% among CM-A, CM-B, and CM-C was significantly similar in their respective comparisons: (1) CM-A & CM-B (*p* = 0.289); (2) CM-A & CM-C (*p* = 0.052); CM-B & CM-C (*p* = 0.112). CM-B and CM-C are similar to each other for both type of cases (*p* = 0.112 non-litigated; *p* = 0.119 litigated).

**Table 5:** Results of Kruskal–Wallis Test with the post hoc test and Bonferroni correction for permanent disability percentage

	Group 1-Group 2	Test statistic	Std. error	Std. test statistic	Sig	Adj. sig.^a^
Non-litigated (*n* = 273; df = 4)	SWIM & Actual	63.250	13.654	4.632	< 0.001	0.000
	SWIM & CM-A	− 77.717	13.654	− 5.692	< 0.001	0.000
	SWIM & CM-B	− 82.511	13.654	− 6.043	< 0.001	0.000
	SWIM & CM-C	− 104.239	13.654	− 7.634	< 0.001	0.000
	Actual & CM-A	− 14.467	13.654	− 1.060	0.289	1.000
	Actual & CM-B	− 19.261	13.654	− 1.411	0.158	1.000
	Actual & CM-C	− 40.989	13.654	− 3.002	0.003	0.027
	CM-A & CM-B	− 4.793	13.654	− 0.351	0.726	1.000
	CM-A & CM-C	− 26.522	13.654	− 1.942	0.052	0.521
	CM-C & CM-B	− 21.728	13.654	− 1.591	0.112	1.000
Litigated (*n* = 273; df = 4)	SWIM & Actual	558.278	30.279	18.438	0.000	0.000
	SWIM & CM-A	− 340.018	30.279	− 11.229	0.000	0.000
	SWIM & CM-B	− 481.178	30.279	− 15.891	0.000	0.000
	SWIM & CM-C	− 511.066	30.279	− 16.878	0.000	0.000
	Actual & CM-A	− 141.161	30.279	− 4.662	< 0.001	0.000
	Actual & CM-B	− 171.048	30.279	− 5.649	< 0.001	0.000
	Actual & CM-C	− 29.888	30.279	− 0.987	0.324	1.000
	CM-A & CM-B	218.260	30.279	7.208	< 0.001	0.000
	CM-A & CM-C	77.099	30.279	2.546	0.011	0.109
	CM-C & CM-B	47.211	30.279	1.559	0.119	1.000

^a^Significance values have been adjusted by Bonferroni correction for multiple tests. Each row tests the nullhypothesis that the Group 1 and Group 2 distributions are the same. Asymptotic significances (2-sided tests) are displayed. The significance level is 0.050

## Other Results

**Fig. 2:**
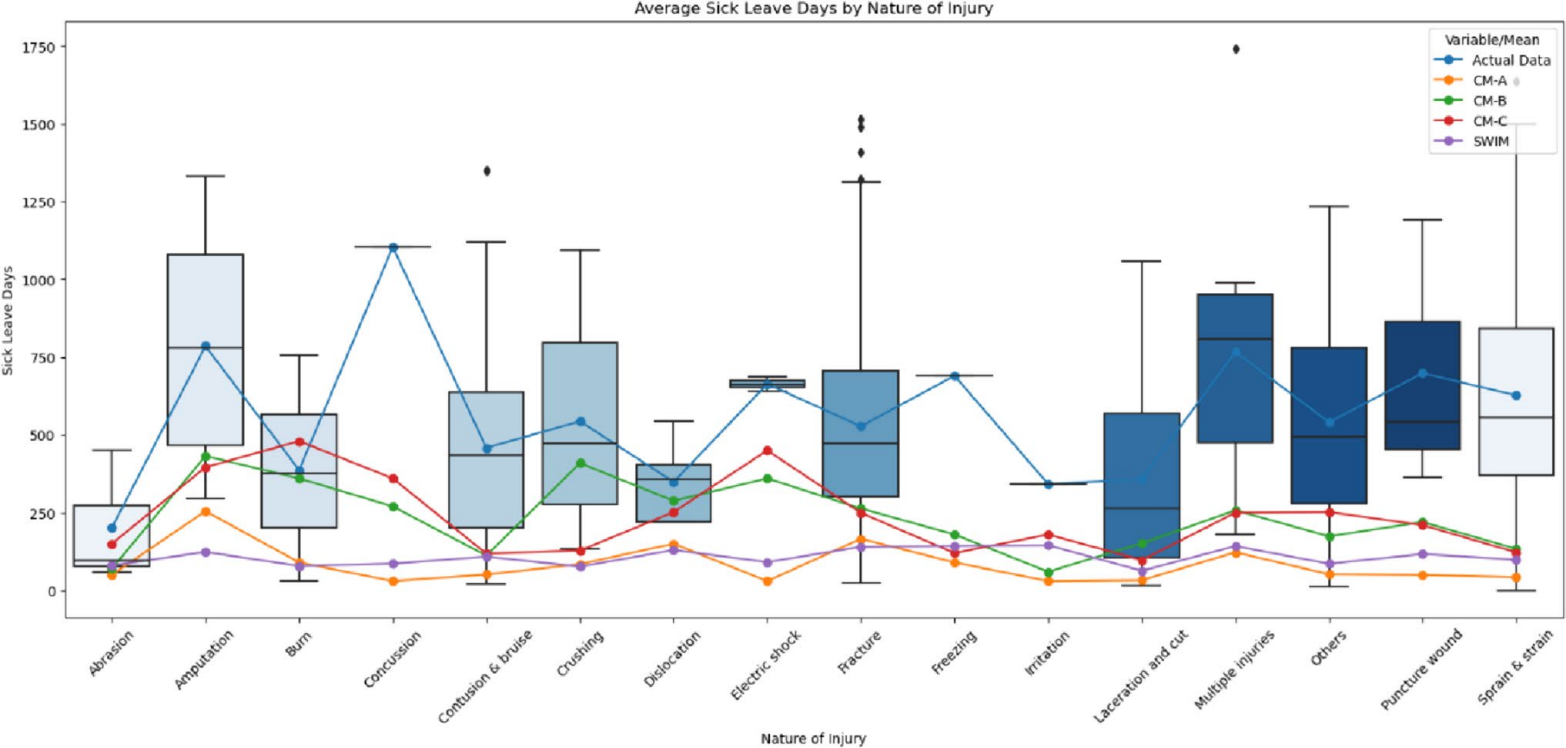
Average sick leave days by nature of injury across actual data, SWIM, CM-A, CM-B, and CM-C

Table 6 presented the average number of sick leave days and the percentage of permanent disability (PD%) by different case types. Additionally, Fig. 2 elucidated that Case Manager B (CM-B) and Case Manager C (CM-C) estimated a higher average number of sick leave days for non-musculoskeletal injuries, such as burns, concussions, or electric shocks, and for severe injuries, including amputations, dislocations, and multiple injuries. The distribution further revealed that the estimations deviated from the actual data in the sequence of CM-C, CM-B, SWIM, and CM-A. The proximity distribution for SWIM and CM-A, as well as CM-B and CM-C, aligned with the findings of this study.

**Table 6:** Mean of sick leave days and permanent disability percentage

	Actual	SWIM	CM-A	CM-B	CM-C
Sick leave (days)					
Non-litigated	271	110	109	190	169
Litigated	648	120	97	225	213
PD (%)					
Non-litigated	3	1	2	2	3
Litigated	6	1	2	3	3

*PD* permanent disability

**Fig. 3:**
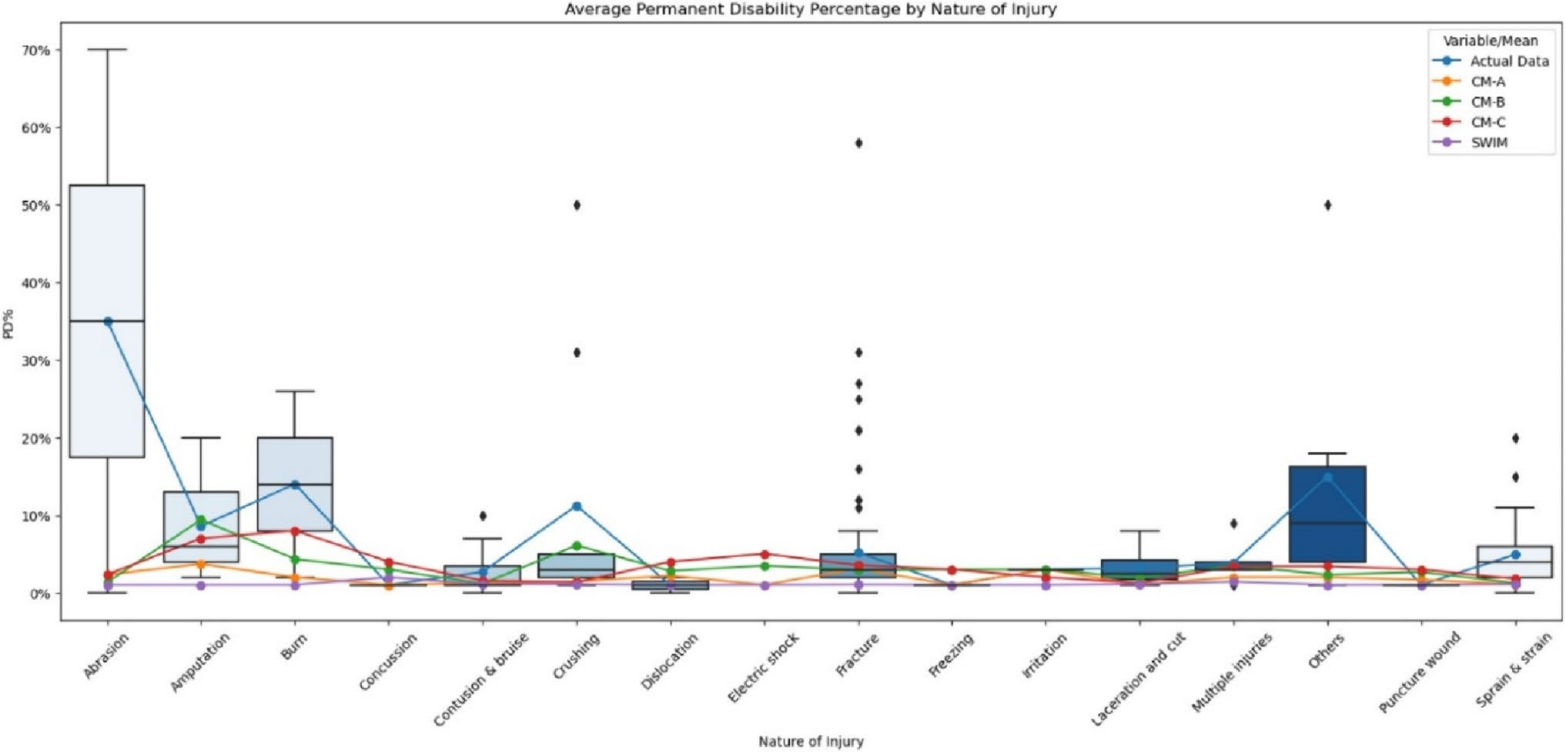
Average permanent disability percentage by nature of injury across actual data, SWIM, CM-A, CM-B, and CM-C

Subsequently, Fig. 3 illustrated that the average PD% predicted by SWIM remained relatively consistent across different types of injuries, corroborating the Kruskal–Wallis test result that it exhibited no similarity across all groups. The boxplot in Fig. 3 was based on the Actual PD%.

## Discussion

The evaluation of the SWIM system's accuracy within this study revealed that its predictions for sick leave days were comparable to those made by Case Manager A (CM-A) for non-litigated cases, with the inter-rater reliability analysis indicating moderate reliability among these comparisons. Moreover, the assessments provided by Case Manager B (CM-B) and Case Manager C (CM-C) showed significant similarity and demonstrated robust reliability for both non-litigated and litigated cases. When it comes to predicting the percentage of permanent disability (PD%), SWIM's forecasts exhibited significant divergence from all groups, when dealing with types of injuries for which the AI has not been sufficiently trained. This discrepancy highlights the importance of a comprehensive training dataset for AI systems like SWIM, ensuring they are well-equipped to accurately predict outcomes across a wide range of injury types. The limitations in SWIM's predictive accuracy for less common or more complex injuries suggest that enhancing the AI's training with a broader spectrum of case types could improve its utility and reliability in clinical decision support. Although the study's generalizability may be impacted by the small number of case managers involved, the findings represent a significant progress in improving the accuracy of CDMS for handling work injury cases in Hong Kong. This advancement is critical for enhancing the support and rehabilitation process for injured workers, contributing to more efficient and effective case management practices in the region.

The similarity between the average number of sick leave days estimated by Case Manager A (CM-A) and SWIM underscores a key outcome of this study: SWIM's predictions of sick leave days were closely aligned with those made by CM-A, who possessed approximately 4 years of case management experience. This correlation was not observed with Case Manager B (CM-B) and Case Manager C (CM-C), who have 9 and 20 years of experience, respectively. All three case managers performed their assessments without prior knowledge of whether a case was non-litigated or litigated, relying solely on the same dataset as SWIM. This approach highlighted a significant variance among the case managers, with CM-A's estimations paralleling SWIM's, whereas CM-B and CM-C's estimates diverged. This disparity may be attributed to several factors, including differences in years of experience, academic backgrounds, and the companies for which they work. A notable factor could be the divergent reserving guidelines adopted by their respective companies. CM-B and CM-C were employed by the same company, likely utilizing a consistent set of guidelines and estimation approaches, whereas CM-A worked for a different firm with unique reserving criteria. Such internal guidelines can vary significantly, affecting the weighting of case information and the overall estimation process. Additionally, the methodology applied in adhering to these guidelines may further contribute to variability in estimates. The observed differences in estimation patterns for non-musculoskeletal injuries between “SWIM and CM-A” versus “CM-B and CM-C” suggest distinct algorithmic approaches or considerations between these groups. Yet, the study does not specify the algorithms or methodologies used by each group. This ambiguity underscores the need for further research to elucidate the various considerations case managers might employ in their initial estimates, particularly for non-musculoskeletal injuries, and to explore the implications of these findings for the development and implementation of AI tools like SWIM in clinical and insurance settings.

Figure 2 further elucidates significant discrepancies in the estimated sick leave days between SWIM, CM-A, CM-B, CM-C, and the actual data. The actual data, derived from cases that have been resolved, were utilized retrospectively to assess the accuracy of SWIM. This indicates that the real cases evolved over a certain period, culminating in a longer duration of sick leave than initially predicted. Such differences imply the influence of various factors throughout the case's progression. Numerous studies have identified that, as cases advance, a variety of psychosocial factors emerge, potentially impacting the trajectory of these cases and leading to prolonged sick leave, thereby affecting the return-to-work outcomes [28–31]. These factors may include cases becoming legal matters [32, 33], the injured employees’ self-perception on employment readiness and maintaining a belief that they were unfit for work [34, 35], developing a compensation-oriented mindset rather than a recovery-oriented [36–39] or adopting a ‘sick role’ such as the Parsonian Model [40]. It is critical to highlight that the case managers in this study made initial sick leave estimations without accounting for psychosocial factors. They were not provided with information regarding the injured employees' self-perceptions about returning to work, nor whether they had legal representation. The case managers relied solely on their judgment, informed by their background and experience, to assess each case. Moreover, the prognosis estimation often hinges on the case manager's experienced judgment. The identification of high-risk factors might alter the trajectory of individual cases as more information is gathered from the injured employee or as the case evolves. A key responsibility of case managers is to develop a case management plan that outlines the direction and frequency of treatment, aiming to facilitate the injured worker's recovery at the minimum possible cost. The training, experience, and access to information of case managers play a significant role in determining the quality of the case management plan [41] and the selection of skilled nurse case managers also helped facilitate the implementation of a successful case management interventions [42]. The other qualitative study from Yanar et al. noted that case managers’ different perceptions of “early” return to work differed the case trajectory despite the agreement on encouraging return to work [43]. Therefore, a case manager’s experience, knowledge, and further case information contents to be obtained has influence on the case prognosis estimation or psychosocial factors identification. Further research is suggested to find out the potential factors that contributing to this identified difference.

It was unexpected that the SWIM’s prediction on PD% were different (*p*  ≤ 0.001) across the 3 case managers. Nevertheless, the estimations made by the three case managers align closely with the actual data for CM-A and CM-B in non-litigated cases, and for CM-C in litigated cases. Figure 3 provides additional support for this assertion. Injuries such as fractures, contusions and bruises, and amputations were among the most accurately predicted types of injury by these groups, excluding SWIM. This observation, in conjunction with the findings related to sick leave days, suggests that the percentage of permanent disability (PD%) estimated by humans might not be significantly influenced by the progression of the case. However, the duration of sick leave may extend as cases progress through various stages of incidents, a conclusion supported by previous research.

It is essential to acknowledge the diversity of factors that case managers, varying in experience and background, might take into account when predicting the prognosis of an injury. Case management embodies a multifaceted process, entwined with a plethora of human elements and disparate responses from injured workers. Given the distinct nature of each work injury case's rehabilitation trajectory, it underscores the importance of ongoing endeavors to emulate human judgment in this domain. Consequently, enhancing the fidelity of simulations to approximate human estimations more closely is highly recommended to improve outcomes in case management.

## Limitations

The development of SWIM provides a valuable tool for clinical decision-making in Hong Kong, particularly for predicting work injury rehabilitation and sick leave days in non-litigated cases. However, there is room for SWIM to improve its accuracy in replicating the expertise of more experienced case managers, thereby addressing the resource shortage in the market. Despite the absence of psychosocial factors in the data provided to case managers, these factors are often considered by case managers due to their historical case handling experience. Given the jurisdiction of Hong Kong, it is common for injured workers to file litigation claims, which typically have a significant impact on return to work and compensation. Therefore, it is recommended that clinical decision-making tools in Hong Kong incorporate psychosocial factors, including litigation risk, when devising treatment plans. Medical treatment outcomes are notoriously poor in patients with pending litigation following disability claims, especially those covered by workers’ compensation programs [44]. Consequently, this study suggests further research on work injury rehabilitation predictions, specifically examining or considering psychosocial factors.

Furthermore, the limited number of case managers involved in this study (*n* = 3) raises concerns regarding the generalizability and scalability of the findings. The modest sample size may not sufficiently represent the wider community of case managers likely to interact with the SWIM system. Case managers hail from varied backgrounds, possess different levels of expertise, and operate within distinct environments, all of which could affect their viewpoints and engagement with the system. Consequently, the system's efficacy and performance might differ when deployed among a more extensive cohort of case managers in real-world scenarios. To corroborate and expand upon the conclusions of this study, future research endeavors should involve a larger and more varied group of case managers.

## Conclusion

Work injury rehabilitation is an issue of international importance, and this study aimed to assess the accuracy of the SWIM system, a clinical decision-support tool, in the context of Hong Kong. This tool assists case managers in forecasting the work disability outcomes of injured workers. The findings indicated that SWIM's estimations were comparable to those made by a case manager with approximately 4 years of case management experience. Nonetheless, to confirm the applicability of these results more broadly, further research involving a larger cohort of case managers is necessary. Additional studies are also recommended to investigate the factors that arise during case progression, which could enhance the accuracy of predictions in comparison with those made by more experienced case managers or against actual case outcomes. Despite these limitations, this study marks a significant advancement for Clinical Decision Management Systems (CDMS) developed in Hong Kong. With the demonstrated success of CDMS in predicting disability outcomes for work injury cases, this technology could address the critical needs for accurate case estimations for reserve allocation and the planning of rehabilitation services for injured employees.

## Data Availability

The datasets generated during and/or analyzed during the current study are available from the corresponding author on reasonable request.
